# Comparison of One-Year auditory rehabilitation outcomes by etiology in pediatric patients with bilateral severe hearing loss (70–90 dB): enlarged vestibular aqueduct vs. Other causes

**DOI:** 10.1007/s00405-025-09649-6

**Published:** 2025-09-18

**Authors:** Seung Jae Lee, Junhyung Bae, Do Hyun Chung, Heonjeong Oh, Jin Hee Han, Yehree Kim, Byung Yoon Choi

**Affiliations:** 1 Department of Otorhinolaryngology-Head and Neck Surgery, Ilsan Paik Hospital, Inje University College of Medicine, Goyang, South Korea; 2 Department of Otorhinolaryngology-Head and Neck Surgery, Seoul National University Bundang Hospital, Seongnam, South Korea; 3 Department of Otorhinolaryngology-Head and Neck Surgery, Seoul National University Hospital, Seoul, South Korea; 4 Department of Otorhinolaryngology-Head and Neck Surgery, Seoul National University College of Medicine, Seoul, South Korea; 5 Sensory Organ Research Institute, Seoul National University Medical Research Center, Seoul, South Korea

**Keywords:** Hearing aids, Cochlear implantation, Conductive hearing loss, Language development, Speech disorders

## Abstract

**Purpose:**

This study aimed to compare short-term language outcomes following hearing aid rehabilitation in pediatric patients with severe bilateral hearing loss (70–90 dB), with a particular focus on differences according to etiology. We hypothesized that children with enlarged vestibular aqueduct (EVA) exhibit more favorable speech development compared to those with other genetic or structural causes of hearing loss, and explored the potential presence of a “hidden” air-bone gap associated with EVA.

**Methods:**

We retrospectively reviewed 36 children under five years of age diagnosed with bilateral severe sensorineural hearing loss and ascertained before age two at Seoul National University Bundang Hospital. Patients were classified into EVA (*n* = 16) and non-EVA (*n* = 20) groups based on radiologic and genetic data. All participants underwent one year of bilateral hearing aid rehabilitation. Speech and language outcomes were assessed using the Categories of Auditory Perception (CAP), Sequenced Language Scale for Infants (SELSI), and Receptive and Expressive Vocabulary Test (REVT), and were compared pre- and post-treatment.

**Results:**

Both groups showed improved CAP scores after one year. However, the EVA group exhibited significantly better expressive language percentile scores (mean 41.8 ± 30.9) compared to the non-EVA group, despite progressive threshold deterioration. Receptive language also improved more in the EVA group, although not statistically significant.

**Conclusion:**

Children with EVA may achieve superior short-term language outcomes with hearing aids, potentially due to a third window–related hidden air-bone gap. However, given the progressive nature of EVA, long-term follow-up is required to assess articulation development and determine optimal timing for cochlear implantation.

**Supplementary Information:**

The online version contains supplementary material available at 10.1007/s00405-025-09649-6.

## Introduction

The importance of rehabilitation for children with severe congenital hearing loss cannot be over-emphasized as early childhood has a critical period for the development of receptive, expressive language, pronunciation, and cognition. Children with auditory deprivation have been shown to demonstrate poorer vocabulary skills and speech development compared to age-matched normal hearing children [1–3]. Commonly referred to as a ‘critical period’ for natural language acquisition, this period is when language is acquired more naturally and accurately and ends during the first year of life [4, 5]. Thus, children who miss this window of opportunity due to bilateral sensorineural hearing loss (SNHL) are expected to represent severe speech and syntactic difficulties later in life.

 Non-syndromic enlarged vestibular aqueduct (EVA) syndrome, known as DFNB4, is manifested with an autosomal recessive inherited hearing loss and one of the most common structural inner ear anomalies in children with permanent hearing loss, constituting up to 12% among children with hearing loss [6, 7]. In conjunction with Pendred syndrome (OMIM#274600), both etiologies are associated with pathogenic SLC26A4 gene variants. Symptoms may include from severe and profound SNHL at birth to milder cases with progressive, fluctuating mixed or SNHL, most likely due to enlarged vestibular aqueducts and endolymphatic sacs [8–10].

 When it comes to bilateral congenital profound hearing loss (over 90dB), the auditory rehabilitation method should be focused on cochlear implantation (CI) as early as possible due to its powerful amplification effect. Previously, a number of studies have documented language outcomes of CI in children with EVA, elucidating that receiving CI at earlier ages has a more positive impact on language development [11–13]. Resultantly, efforts to minimize the time gap between the period when children fulfill candidacy criteria for CI and actual implantation is pivotal to ensure continuous optimal access to spoken language and reduce the potential impact of sudden hearing loss in deafened children with EVA [14]. However, there arises a controversy whether pediatric patients with auditory thresholds limited to severe degree bilaterally (70 ~ 90dB) do really need and undergo CI, albeit of having language performance close to or exceeds above the average speech levels of normal hearing children.

 Interestingly, for pediatric patients with severe bilateral SNHL, linguistic performance measured with speech evaluation tests seem to differ according to the etiology of hearing loss. That is, for the patients with same degree of hearing loss, patients with EVA due to SLC26A4 variants seem to acquire better linguistic performance when comparing the speech evaluation test data at the time of diagnosis and after a year of hearing rehabilitation than those with other etiologies. Given this, the goal of this study is to compare the speech evaluation test results between pediatric patients with EVA and other etiologies, and if EVA patients have better linguistic performance than patients with other etiologies with the same degree of hearing loss.

## Materials and methods

### Study participants

 We retrospectively reviewed the medical records of 38 pediatric patients (23 boys and 15 girls) who visited outpatient clinic between July 2018 and April 2022 at Seoul National University Bundang Hospital (SNUBH). Inclusion criteria included: (1) children younger than 5 years of age who showed bilateral severe hearing loss (70 ~ 90 dB), (2) those who had been diagnosed with bilateral EVA on temporal bone computed tomography (TBCT) scans or (3) diagnosed with DFNB4 due to SLC26A4 variants after genetic testing. Basic demographic (age, sex, age at diagnosis of hearing loss, and age when starting to wear hearing aids), audiological, genotypic, and radiological data have also been investigated. Pediatric patients diagnosed with global developmental delay and/or cognitive delay as evaluated by Bayley Scales of Infant and Toddler Development Screening Test (BSID version 4) [15] were excluded in this study. This study was approved by the Institutional Review Board of SNUBH (IRB no. B-2406-908-103), and the informed patient consent was waived due to the retrospective design of the chart review. All protocols and procedures were conducted in accordance with the Declaration of Helsinki.

### Audiological evaluation

Audiological evaluations were conducted with auditory brainstem response (ABR) test and auditory steady-state response (ASSR) by licensed professional audiologists at SNUBH. A conventional pure tone audiometry, including air and bone conduction thresholds, was not performed in most of the pediatric cohort due to patients’ relatively young ages and poor cooperation. According to our institution’s diagnostic and therapeutic pipelines, decision of when to apply hearing aid was determined according to two main criteria: (1) when the average of ABRT and/or ASSR thresholds was limited to bilateral severe HL (70~90dB), and/or (2) when a receptive score of speech evaluation test was delayed less than 5 months compared to that of normal children of equivalent age. When the development of receptive speech performance was not sufficient enough even after one year of auditory rehabilitation (i.e. when the receptive language got delayed by more than 5 months compared to the age-matched control group) and/or when the auditory thresholds exceeded over 90dB, CI was considered.

### Speech evaluation

Speech evaluation was conducted mainly based on three assessment tools. First, the Categories of Auditory Performance (CAP) score, a hierarchical scale ranging from 0 to 7, was used to evaluate auditory perceptual abilities, where higher levels indicate better auditory performance (e.g., Level 0: no awareness of environmental sounds; Level 7: use of the telephone with a familiar speaker; Supplementary Table 1) [16, 17]. The primary assessment of vocabulary skills was measured with the Sequenced Language Scale for Infants (SELSI) and the Receptive and Expressive Vocabulary Test (REVT). SELSI and REVT are both standardized, validated Korean tools for assessing receptive and expressive language abilities. Specifically, the SELSI has been developed to assess the receptive and expressive language abilities of 4 to 35 months through parental reports, while the REVT has been designed to assess the levels of vocabulary development of healthy children and adolescents without any learning difficulties, ranging from 2 to 18 years old [18, 19]. In summary, these assessments provide percentile scores based on chronological age norms, allowing clear and precise evaluation of language development levels compared to peers. In this study, the REVT was administered as an alternative tool when a child was aged 2 years or older.

To determine the age-appropriate level and status of vocabulary (speech) development, the raw scores were transformed and compared with age-adjusted percentile scores. All of the speech evaluation tests were administered by certified speech-language pathologists at SNUBH before and one year after treatment.

### Statistical analysis

 All the data are presented as mean ± standard deviation (SD). Statistical analyses were conducted using SPSS software for Windows version 25.0 (IBM Corp., Armonk, NY, USA) and visualized using the GraphPad Prism software (version 9.0.0; GraphPad Software Inc., San Diego, CA, USA). Normality of the data was assessed using the Shapiro–Wilk test. As the data did not follow a normal distribution, non-parametric statistical methods were applied. Specifically, the Wilcoxon signed-rank test was used instead of a 2 × 2 mixed ANOVA to analyze within-group differences over time. CAP scores and percentiles of receptive and expressive SELSI and REVT tests were compared pairwise between the two groups at pre- and post-treatment period with Mann-Whitney U tests. A p-value of less than 0.05 was reported as statistically significant.

## Results

### Overall demographic characteristics

 A total of thirty-six pediatric patients were enrolled in this study, and the cohort was classified into EVA (N = 16) and other etiologies (non-EVA) group (N = 20) according to the genetic etiology (Supplementary Tables 2 and Table 3, respectively). The mean age at initial diagnosis was 4.6 ± 2.3 months for EVA group and 6.9 ± 8.9 months for patients with other etiologies. For the EVA group, all pediatric patients exhibited bilateral EVA and severe SNHL. With respect to the etiologic configurations within the non-EVA group, congenital cytomegalovirus (cCMV) infection and MYO15A variants constituted the largest number, each with 4 patients, followed by 3 patients with DFNB1 due to GJB2 variants, 2 patients with inner ear anomaly (incomplete partition type III) and unknown genetic causes, and each one patient with 4q31.3qter deletion, auditory neuropathy spectrum disorder, EYA1 variant, POU3F4 variant, and TMPRSS3 variant.

All patients underwent at least one year of auditory rehabilitation with bilateral hearing aids after diagnosis. The mean age at the initiation of hearing aid use was 4.6 ± 2.3 and 6.9 ± 8.9 months for EVA and non-EVA group, respectively (ranging from birth to 56 months of age). Among the 36 patients who underwent hearing aid rehabilitation for one year, a total of 21 patients continued with hearing aid rehabilitation beyond the first year (9 in the EVA and 12 in the non-EVA group). Conversely, sixteen patients who experienced further hearing deterioration or failed to achieve satisfactory speech development underwent unilateral or bilateral CI, with 8 patients belonging to the EVA group and the other 8 to the non-EVA group.

### Natural progress of audiological status according to the etiology

 When comparing the ABR thresholds of better ears, both groups showed no statistically significant difference at the pre-treatment period (p-value = 0.895, Mann-Whitney U test). The mean threshold of the EVA group worsened by 6.2 dB—greater than that of the non-EVA group—though the difference was not statistically significant (p-value = 0.146; Mann-Whitney U test) (Table 1).

**Table 1:** The average of hearing thresholds, CAP, receptive, and expressive scores before and after treatment (auditory rehabilitation with hearing aid) for each group are demonstrated. For group comparisons, a Mann Whitney U-test was performed. A *p*-value of less than 0.05 was considered statistically significant

		EVA group (*N* = 16)	Non-EVA group (*N* = 20)	*p*-value
Age at initial treatment (month)		14.1 ± 7.5	20.3 ± 18.7	0.741
Hearing thresholds (better side, dB)	Pre-treatment	76.1 ± 7.1	75.1 ± 5.7	0.912
	Post-treatment	82.3 ± 13.2	74.6 ± 12.8	0.149
CAP score	Pre-treatment	2.6 ± 1.5	3.0 ± 1.7	0.560
	Post-treatment	4.3 ± 1.2	4.4 ± 1.1	0.888
Receptive score (percentile)	Pre-treatment	23.4 ± 22.6	24.1 ± 31.9	0.718
	Post-treatment	33.3 ± 32.3	27.9 ± 30.1	0.440
Expressive score (percentile)	Pre-treatment	26.0 ± 22.1	27.9 ± 28.1	0.912
	Post-treatment	41.8 ± 30.9	20.4 ± 28.8	0.003

Abbreviations: *EVA* enlarged vestibular aqueduct, *CAP* categories of auditory performance

### Speech performances between the EVA and non-EVA group

**Fig. 1:**
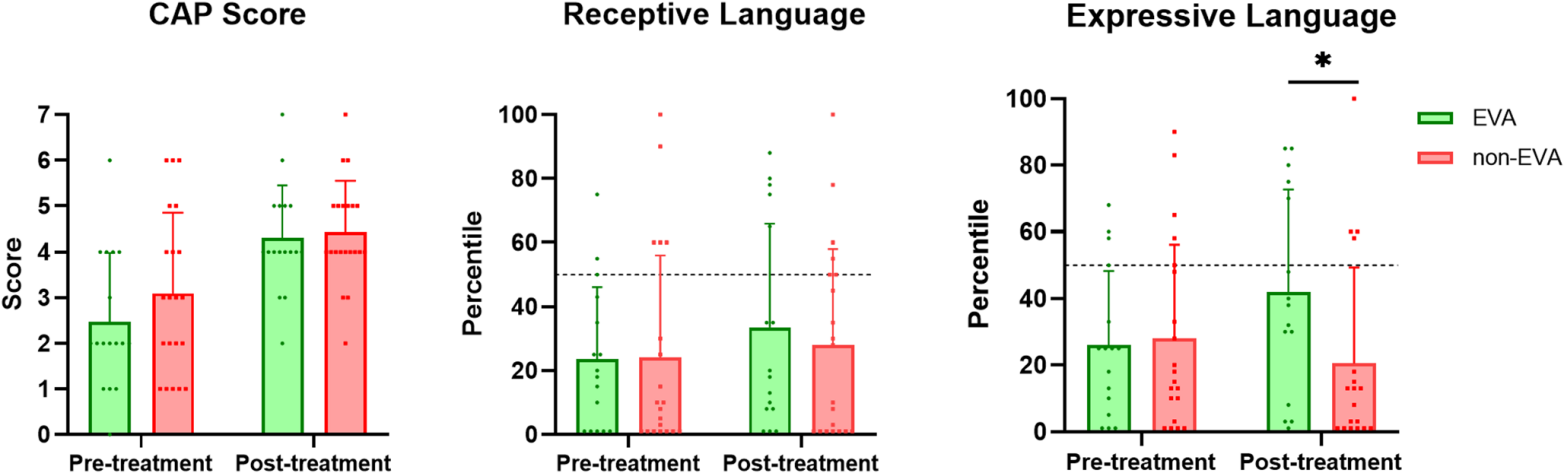
A comparison of each speech performance results between pre- and post-treatment is indicated. Only the expressive language outcomes of the EVA group exhibited significantly better results compared to those of the non-EVA group after one year of hearing aid rehabilitation. A Wilcoxon signed rank test was performed between pre- and post- treatment period and a *p*-value of less than 0.05 was reported as statistically significant

 When comparing the CAP scores, both groups showed improvement after one year of hearing aid use. And no significant differences in CAP scores were found between the two groups at pre- and post-treatment periods (Fig. 1, Supplementary Table 4). For receptive language, the degree of improvement was greater in the EVA group compared to the non-EVA group, although this difference did not reach statistical significance. In terms of expressive language, only EVA group almost caught up the development of normal children even with hearing aid rehabilitation (41.8 ± 30.9 percentile), while the non-EVA group showed a decrease in the average outcome values. Interestingly, the EVA group demonstrated significantly better expressive language outcomes compared to the non-EVA group after one year of hearing aid rehabilitation (Fig. 1, Supplementary Table 4).

## Discussion

Recently, newborn children are newly diagnosed with congenital hearing loss at increasingly younger ages, probably due to the introduction of newborn hearing screening systems. The earlier detection of SNHL has also highlighted the early implementation of auditory rehabilitation with either hearing aids or CI to prevent inevitable developmental or speech delays. The decision-making profiles to proceed auditory rehabilitation with hearing aids or CI in children with congenital hearing loss usually depend on the degree of hearing loss and/or the level of language development. Particularly, even in cases of severe SNHL that may necessitate urgent CI, if the language development is satisfactory within normal range, auditory rehabilitation with hearing aids with rigorous speech therapy might be prioritized instead of CI. In this regard, the present study revealed that in children who are diagnosed with EVA with severe hearing loss (70 ~ 90dB), auditory rehabilitation using hearing aids alone has shown effective language development outcomes compared to those with other etiologies.

EVA is one of the most common radiologic findings in pediatric patients presenting with unilateral or bilateral hearing loss and closely associated with DFNB4 due to *SLC26A4* variant [20]. Among various types of hearing loss, SNHL is the predominant form of hearing loss in patients with EVA, though as demonstrated in other studies, conductive hearing loss confined within low frequencies can be encountered as well [20, 21]. Although the exact mechanism of conductive hearing loss in EVA has not yet been fully clarified, impaired stapes mobility (i.e. stapes fixation) and cochlear mechanics, or an abnormal intracochlear fluid pressure resulting from the third window phenomenon are recognized as possible causes of conductive hearing impairment [22, 23]. The third window abnormality was first described by Minor et al in 1998, indicating anatomical defects in the integrity of the bony structure of the inner ear [24]. Various conditions including EVA, semicircular canal dehiscence, or abnormal bony wall thinning create redundant defects in the bony labyrinth and lead to hydrodynamic third windows [25]. These anatomical abnormalities create the third window phenomenon, resulting in decreased air conduction (AC) and increased bone conduction (BC) threshold, so called “low-frequency air-bone gap [26].” Specifically, a decrease in the pressure gradient across the basilar membrane reduces sound perception, lowering the air conduction (AC) threshold. Meanwhile, if the movement of the round window on the scala tympani side remains unchanged, the pressure difference across the basilar membrane increases, enhancing bone conduction (BC) and improving sound perception [25]. Plus, changes in acoustic soundwave pressure enhance bone conduction, inducing supra-normal bone conduction responses [27].

Although the primary treatment for conductive hearing loss is surgical intervention, there exists an advantage in applying hearing aids as an alternative rehabilitation strategy, when conductive hearing loss is not fully identified. In this study, we hypothesized that the EVA group would exhibit a “hidden” air-bone gap due to the third window phenomenon, and their speech outcomes after one year of auditory rehabilitation with hearing aids would rather exhibit better performance when compared with the non-EVA group. Despite the deterioration of hearing thresholds at post-1-year treatment period, the average speech developmental level of the EVA group, as evidenced by SELSI and REVT percentiles and CAP scores, outperformed when compared with the non-EVA group. Additionally, the EVA group showed relatively higher percentile scores in speech performance (both receptive and expressive) than those matched to their auditory status, measured by ABRT and/or ASSR. In this context, this study has several meaningful implications. First, we identified, for the first time, the genetic causes of severe hearing loss (70–90 dB) in young children, and as expected, *SLC26A4 *variants constituted the largest portion of molecular etiology of pediatric patients exhibiting hearing loss within the specified range. Second, we demonstrated that auditory rehabilitation with hearing aids not only mitigated the adverse effects of progressive hearing deterioration in the EVA group but also led to a significant improvement in both receptive and expressive language percentile scores over one year. Notably, despite more severe hearing loss, the EVA group showed statistically superior expressive language outcomes compared to the non-EVA group, approaching average performance levels. In contrast, the non-EVA group maintained their receptive language percentile scores but exhibited a decline in expressive language percentile rankings during the same period. Our results are in line with those from a recent study by Mey et al. which advocated the advantageous effect of hearing aid rehabilitation in patients with Pendred syndrome and non-syndromic EVA, demonstrating no significant differences in receptive language acquisition levels among those with conventional hearing aids and with CI [28]. Plus, considering the unique audiological characteristic of EVA, as evidenced by the preservation of residual hearing and sustained speech performances [29, 30], the auditory rehabilitation with hearing aids alone would be powerful enough to overcome ‘pseudo-conductive hearing loss’ and achieve adequate language development in children diagnosed with EVA. Our findings strongly support the presence of the hidden air-bone gap, indicating that their actual bone conduction threshold level would have been better than what was actually measured by air conduction ABR or ASSR. Third, although conventional pure-tone audiometry often fails to reveal a significant air-bone gap in EVA cases, it has been proposed that fluctuating endolymphatic pressure or third-window effects can cause a functional air-bone gap that is not readily detected [26, 31]. This hidden air-bone gap may lead to underestimation of conductive components in hearing loss, making patients appear to have pure SNHL. Consequently, hearing aids may provide greater benefits in the EVA group by compensating for this unrecognized conductive component, thereby improving speech audibility and clarity.

 Notably, although both groups showed comparable improvements in auditory perception—as reflected by increased CAP scores after one year of hearing aid use—no statistically significant difference was found between the groups. This suggests that while hearing aid rehabilitation enhances basic auditory performance in both populations, the expressive language gains in the EVA group may reflect additional benefits from correcting a hidden conductive component. However, the lack of a significant difference in receptive language scores between the groups may represent a limitation of the current study, possibly due to the small sample size. Furthermore, it remains unclear why only expressive language scores improved significantly in the EVA group, while receptive scores did not show a parallel trend. This discrepancy highlights the need for further research to better understand the differential impact of hearing rehabilitation on various aspects of language development in children with different etiologies of hearing loss. Additionally, the hypothesis of a functional air-bone gap was inferred indirectly from the speech and language outcomes rather than supported by objective audiological or radiologic data. Future studies incorporating detailed bone conduction thresholds and high-resolution temporal bone imaging will be essential to more directly validate the proposed mechanism and clarify the role of third-window effects in auditory rehabilitation outcomes.

 Our findings suggest that even among pediatric patients with comparable degrees of severe hearing loss, the outcomes of hearing aid use and language rehabilitation may vary significantly depending on the underlying etiology. Specifically, the timing of transition from hearing aid–based rehabilitation to cochlear implantation may differ according to etiology, even within the same threshold range of hearing loss. For instance, patients in the non-EVA group are more likely to undergo cochlear implantation earlier than those in the EVA group under similar audiological conditions. Supporting this, Lee et al. [32] reported that, despite the high prevalence of EVA, relatively few EVA patients received cochlear implants before the age of one. Moreover, considering that a considerable number of EVA patients undergo CI during adolescence or early adulthood [33], it is plausible that EVA patients exhibit a more favorable response to hearing aids compared to those with other etiologies.

 However, the findings of our current study should not be interpreted as indicating that pediatric EVA patients within the severe hearing loss range can achieve complete auditory and language development solely through hearing aid use. In fact, children with this degree of hearing loss often experience progressive deterioration over time, and despite achieving near-normal receptive and expressive language development with hearing aid–based rehabilitation, many eventually undergo CI due to persistent delays in the development of speech articulation [17]. At our institution, CI is performed not only based on language assessment results or hearing levels but also when articulation is poor relative to overall language abilities. In other words, even in cases where there is no significant difference in expressive and receptive language, CI may still be performed if articulation development is inadequate. Given that the evaluation of pronunciation and articulation accuracy is generally feasible only after 26 months of age, these aspects could not be assessed during the follow-up period in the current study cohort, particularly within the EVA group. Therefore, although our study demonstrates favorable short-term language outcomes with hearing aids in EVA patients with severe hearing loss, longer-term follow-up is warranted to determine the sustainability of these outcomes and the eventual need for CI.

## Conclusion

This study highlights the differential impact of etiology on auditory rehabilitation outcomes by hearing aids in pediatric patients with severe hearing loss ranging from 70 to 90 dB who were ascertained before the age of two. Specifically, despite the risk of progressive hearing deterioration and sudden declines in auditory thresholds, children with EVA demonstrated significantly better short-term expressive language development following one year of hearing aid use compared to those with non-EVA etiologies. However, given the progressive nature of EVA-associated hearing loss and the inability to assess articulation development within the study timeframe, these findings should not be interpreted as evidence that rehabilitation by hearing aids alone suffice long-term. Extended follow-up is essential to determine the eventual need for CI, particularly in identifying the threshold level or time point at which CI becomes necessary.

## Supplementary Information

Below is the link to the electronic supplementary material.

Supplementary File 1(DOCX 12.9 KB)

Supplementary File 2(DOCX 23.3 KB)

Supplementary File 3(DOCX 24.2 KB)

Supplementary File 4(DOCX 16.3 KB)
