# Supplementary material for: Comparison of One-Year auditory rehabilitation outcomes by etiology in pediatric patients with bilateral severe hearing loss (70–90 dB): enlarged vestibular aqueduct vs. Other causes
Source: Eur Arch Otorhinolaryngol. 2025 Sep 18;283(1):149–56. doi: 10.1007/s00405-025-09649-6 (PMC12904901; doi:10.1007/s00405-025-09649-6)
Supplement: Supplementary file 1 — (DOCX 12.9 KB) [file 405_2025_9649_MOESM1_ESM.docx]

**Supplementary table 1.** The Categories of Auditory Performance (CAP) criteria.

| Rating | Criterion |
| --- | --- |
| 7 | Uses the telephone with a known listener |
| 6 | Understands conversation without lip-reading |
| 5 | Understands common phrases without lip-reading |
| 4 | Discriminates some speech sounds without lip-reading |
| 3 | Identifies environmental sounds |
| 2 | Responds to speech sounds |
| 1 | Is aware of environmental sounds |
| 0 | Has no awareness of environmental sounds |

DOI: 10.1371/journal.pone.0081568
