# Supplementary material for: Comparison of One-Year auditory rehabilitation outcomes by etiology in pediatric patients with bilateral severe hearing loss (70–90 dB): enlarged vestibular aqueduct vs. Other causes
Source: Eur Arch Otorhinolaryngol. 2025 Sep 18;283(1):149–56. doi: 10.1007/s00405-025-09649-6 (PMC12904901; doi:10.1007/s00405-025-09649-6)
Supplement: Supplementary file 2 — (DOCX 23.3 KB) [file 405_2025_9649_MOESM2_ESM.docx]

**Supplementary table 2**. Details of genotypes within the EVA group.

| **Sample ID** | **Variants of the *SLC26A4* gene** | | **Inheritance** | **Variant genomic position (GRCh37/hg19)**  **dbSNP ID (dbSNP v151)** | **Zygosity** | **In-silico algorithm** | | **Alternative allele frequency** | | **Classification of pathologic variants according to ACMG/AMP guideline** |
| --- | --- | --- | --- | --- | --- | --- | --- | --- | --- | --- |
|  | **Nucleotide change [NM_000441.2]** | **Amino acid change [NP_000432.1]** |  |  |  | **CADD Phred** | **REVEL** | **GMAF** | **KOVA** |  |
| SB747-2159 | c.919-2A>G |  | AR | Chr7:107323898 A>G rs111033313 | heterozygote | 28.40 | NA | G=0.0000664 (93/1401034, GnomAD_exomes) G=0.000194 (29/149320, GnomAD_genomes)  G=0.000306 (37/121000, ExAC) G=0.00062 (48/77418, 38KJPN) | G=0.0010616 (4/3768, KOVA) | **Pathogenic :** PVS1, PS1, PM2, PM3_VeryStrong, PP1_Strong, PP4 |
|  | c.2168A>G | p.His723Arg |  | Chr7:107350577 A>G rs121908362 | heterozygote | 25.30 | 0.933 | G=0.000060 (9/149344, GnomAD_genomes) G=0.000124 (15/121166, ExAC) G=0.00258 (200/77444, 38KJPN) | G=0.0047295 (50/10572, KOVA) | **Pathogenic :** PM2, PM3_VeryStrong, PP1_Strong, PP3, PP4 |
| SB747-1324 | c.919-2A>G |  | AR | Chr7:107323898 A>G rs111033313 | heterozygote | 28.40 | NA | G=0.0000664 (93/1401034, GnomAD_exomes) G=0.000194 (29/149320, GnomAD_genomes)  G=0.000306 (37/121000, ExAC) G=0.00062 (48/77418, 38KJPN) | G=0.0010616 (4/3768, KOVA) | **Pathogenic :** PVS1, PS1, PM2, PM3_VeryStrong, PP1_Strong, PP4 |
|  | c.2168A>G | p.His723Arg |  | Chr7:107350577 A>G rs121908362 | heterozygote | 25.30 | 0.933 | G=0.000060 (9/149344, GnomAD_genomes) G=0.000124 (15/121166, ExAC) G=0.00258 (200/77444, 38KJPN) | G=0.0047295 (50/10572, KOVA) | **Pathogenic :** PM2, PM3_VeryStrong, PP1_Strong, PP3, PP4 |
| SB479-925 | c.919-2A>G |  | AR | Chr7:107323898 A>G rs111033313 | homozygote | 28.40 | NA | G=0.0000664 (93/1401034, GnomAD_exomes) G=0.000194 (29/149320, GnomAD_genomes)  G=0.000306 (37/121000, ExAC) G=0.00062 (48/77418, 38KJPN) | G=0.0010616 (4/3768, KOVA) | **Pathogenic :** PVS1, PS1, PM2, PM3_VeryStrong, PP1_Strong, PP4 |
| SB681-1218 | c.2168A>G | p.His723Arg | AR | Chr7:107350577 A>G rs121908362 | homozygote | 25.30 | 0.933 | G=0.000060 (9/149344, GnomAD_genomes) G=0.000124 (15/121166, ExAC) G=0.00258 (200/77444, 38KJPN) | G=0.0047295 (50/10572, KOVA) | **Pathogenic :** PM2, PM3_VeryStrong, PP1_Strong, PP3, PP4 |
| SB848-1454 | c.2168A>G | p.His723Arg | AR | Chr7:107350577 A>G rs121908362 | homozygote | 25.30 | 0.933 | G=0.000060 (9/149344, GnomAD_genomes) G=0.000124 (15/121166, ExAC) G=0.00258 (200/77444, 38KJPN) | G=0.0047295 (50/10572, KOVA) | **Pathogenic :** PM2, PM3_VeryStrong, PP1_Strong, PP3, PP4 |
| SB1380-2160 | c.919-2A>G |  | AR | Chr7:107323898 A>G rs111033313 | heterozygote | 28.40 | NA | G=0.0000664 (93/1401034, GnomAD_exomes) G=0.000194 (29/149320, GnomAD_genomes)  G=0.000306 (37/121000, ExAC) G=0.00062 (48/77418, 38KJPN) | G=0.0010616 (4/3768, KOVA) | **Pathogenic :** PVS1, PS1, PM2, PM3_VeryStrong, PP1_Strong, PP4 |
|  | c.2168A>G | p.His723Arg |  | Chr7:107350577 A>G rs121908362 | heterozygote | 25.30 | 0.933 | G=0.000060 (9/149344, GnomAD_genomes) G=0.000124 (15/121166, ExAC) G=0.00258 (200/77444, 38KJPN) | G=0.0047295 (50/10572, KOVA) | **Pathogenic :** PM2, PM3_VeryStrong, PP1_Strong, PP3, PP4 |
| SB824-1428 | c.2168A>G | p.His723Arg | AR | Chr7:107350577 A>G rs121908362 | homozygote | 25.30 | 0.933 | G=0.000060 (9/149344, GnomAD_genomes) G=0.000124 (15/121166, ExAC) G=0.00258 (200/77444, 38KJPN) | G=0.0047295 (50/10572, KOVA) | **Pathogenic :** PM2, PM3_VeryStrong, PP1_Strong, PP3, PP4 |
| SB1381-2161 | c.919-2A>G |  | AR | Chr7:107323898 A>G rs111033313 | heterozygote | 28.40 | NA | G=0.0000664 (93/1401034, GnomAD_exomes) G=0.000194 (29/149320, GnomAD_genomes)  G=0.000306 (37/121000, ExAC) G=0.00062 (48/77418, 38KJPN) | G=0.0010616 (4/3768, KOVA) | **Pathogenic :** PVS1, PS1, PM2, PM3_VeryStrong, PP1_Strong, PP4 |
|  | c.1229C>T | p.Thr410Met |  | Chr7:107330648 C>T rs111033220 | heterozygote | 31.00 | 0.907 | T=0.000054 (8/149230, GnomAD_genomes) T=0.000190 (23/121222, ExAC) T=0.00043 (33/77444, 38KJPN) | T=0.00037951 (4/10540, KOVA) | **Pathogenic :** PM3_VeryStrong, PM5, PP1_Strong, PP3, PP4 |
| SB868-1482 | c.2168A>G | p.His723Arg | AR | Chr7:107350577 A>G rs121908362 | homozygote | 25.30 | 0.933 | G=0.000060 (9/149344, GnomAD_genomes) G=0.000124 (15/121166, ExAC) G=0.00258 (200/77444, 38KJPN) | G=0.0047295 (50/10572, KOVA) | **Pathogenic :** PM2, PM3_VeryStrong, PP1_Strong, PP3, PP4 |
| SB1382-2162 | c.2168A>G | p.His723Arg | AR | Chr7:107350577 A>G rs121908362 | homozygote | 25.30 | 0.933 | G=0.000060 (9/149344, GnomAD_genomes) G=0.000124 (15/121166, ExAC) G=0.00258 (200/77444, 38KJPN) | G=0.0047295 (50/10572, KOVA) | **Pathogenic :** PM2, PM3_VeryStrong, PP1_Strong, PP3, PP4 |
| SB568-1046 | c.2168A>G | p.His723Arg | AR | Chr7:107350577 A>G rs121908362 | homozygote | 25.30 | 0.933 | G=0.000060 (9/149344, GnomAD_genomes) G=0.000124 (15/121166, ExAC) G=0.00258 (200/77444, 38KJPN) | G=0.0047295 (50/10572, KOVA) | **Pathogenic :** PM2, PM3_VeryStrong, PP1_Strong, PP3, PP4 |
| SB769-1354 | c.2168A>G | p.His723Arg | AR | Chr7:107350577 A>G rs121908362 | homozygote | 25.30 | 0.933 | G=0.000060 (9/149344, GnomAD_genomes) G=0.000124 (15/121166, ExAC) G=0.00258 (200/77444, 38KJPN) | G=0.0047295 (50/10572, KOVA) | **Pathogenic :** PM2, PM3_VeryStrong, PP1_Strong, PP3, PP4 |
| SB1383-2163 | c.2168A>G | p.His723Arg | AR | Chr7:107350577 A>G rs121908362 | homozygote | 25.30 | 0.933 | G=0.000060 (9/149344, GnomAD_genomes) G=0.000124 (15/121166, ExAC) G=0.00258 (200/77444, 38KJPN) | G=0.0047295 (50/10572, KOVA) | **Pathogenic :** PM2, PM3_VeryStrong, PP1_Strong, PP3, PP4 |
| SB1134-1848 | c.2027T>A | p.Leu676Gln | AR | Chr7:107342495 T>A rs111033318 | heterozygote | 27.50 | 0.733 | A=0.0000014 (2/1393316, GnomAD_exomes) | A=0.00018936 (2/10562, KOVA) | **Pathogenic :** PM2, PM3_VeryStrong, PM5, PP1_Strong, PP3, PP4 |
|  | c.2168A>G | p.His723Arg |  | Chr7:107350577 A>G rs121908362 | heterozygote | 25.30 | 0.933 | G=0.000060 (9/149344, GnomAD_genomes) G=0.000124 (15/121166, ExAC) G=0.00258 (200/77444, 38KJPN) | G=0.0047295 (50/10572, KOVA) | **Pathogenic :** PM2, PM3_VeryStrong, PP1_Strong, PP3, PP4 |
| SB1384-2164 | c.2162C>T | p.Thr721Met | AR | Chr7:107350571 C>T rs121908363 | heterozygote | 29.60 | 0.864 | T=0.000027 (4/149208, GnomAD_genomes) T=0.000058 (7/121120, ExAC) T=0.00010 (8/77444, 38KJPN) | T=0.00018918 (2/10572, KOVA) | **Pathogenic :** PM2, PM3_VeryStrong, PM5, PP1_Strong, PP3, PP4 |
|  | c.2168A>G | p.His723Arg |  | Chr7:107350577 A>G rs121908362 | heterozygote | 25.30 | 0.933 | G=0.000060 (9/149344, GnomAD_genomes) G=0.000124 (15/121166, ExAC) G=0.00258 (200/77444, 38KJPN) | G=0.0047295 (50/10572, KOVA) | **Pathogenic :** PM2, PM3_VeryStrong, PP1_Strong, PP3, PP4 |
| SB599-1097 | c.2168A>G | p.His723Arg | AR | Chr7:107350577 A>G rs121908362 | homozygote | 25.30 | 0.933 | G=0.000060 (9/149344, GnomAD_genomes) G=0.000124 (15/121166, ExAC) G=0.00258 (200/77444, 38KJPN) | G=0.0047295 (50/10572, KOVA) | **Pathogenic :** PM2, PM3_VeryStrong, PP1_Strong, PP3, PP4 |

Abbreviations: AR, autosomal recessive; GMAF, global minor allele frequency; NA, not available; ACMG, the American College of Medical Genetics and Genomics; AMP, the Association for Molecular Pathology.

Web resources: CADD: Combined Annotation Dependent Depletion (https://cadd.gs.washington.edu/),REVEL: Rare Exome Variant Ensemble Learner (https://sites.google.com/site/revelgenomics/),KOVA: Korean Variant Archive for a reference database of genetic variations in the Korean population (https://www.kobic.re.kr/kova/) ,gnomAD: The Genome Aggregation Database (https://gnomad.broadinstitute.org/).
