# Supplementary material for: Comparison of One-Year auditory rehabilitation outcomes by etiology in pediatric patients with bilateral severe hearing loss (70–90 dB): enlarged vestibular aqueduct vs. Other causes
Source: Eur Arch Otorhinolaryngol. 2025 Sep 18;283(1):149–56. doi: 10.1007/s00405-025-09649-6 (PMC12904901; doi:10.1007/s00405-025-09649-6)
Supplement: Supplementary file 3 — (DOCX 24.2 KB) [file 405_2025_9649_MOESM3_ESM.docx]

**Supplementary table 3**. Details of genotypes within the non-EVA group.

| **Sample ID** | **Causative variants**  **(if identified)** | | | **Inheritance** | **Variant genomic position (GRCh37/hg19)**  **dbSNP ID (dbSNP v151)** | **Zygosity** | **In-silico algorithm** | | **Alternative allele frequency** | | **Classification of pathologic variants according to ACMG/AMP guideline** |
| --- | --- | --- | --- | --- | --- | --- | --- | --- | --- | --- | --- |
|  | **Gene** | **Nucleotide change** | **Amino acid change** |  |  |  | **CADD Phred** | **REVEL** | **GMAF** | **KOVA** |  |
| SB1386-2166 | *GJB2* | [NM_004004.5] c.235del | [NP_003995.2] p.Leu79Cys*fs*Ter3 | AR | Chr13:20763485 AG>A rs80338943 | homozygote | 34.00 | NA | delG=0.0001834 (257/1401430, GnomAD_exomes) delG=0.000308 (46/149288, GnomAD_genomes) | delG=0.0065 (47/7234, Korea) | Pathogenic : PVS1, PM3_Very Strong, PS3_Moderate, BS1 |
| SB683-1221 | *GJB2* | [NM_004004.5] c.235del | [NP_003995.2] p.Leu79Cys*fs*Ter3 | AR | Chr13:20763485 AG>A rs80338943 | homozygote | 34.00 | NA | delG=0.0001834 (257/1401430, GnomAD_exomes) delG=0.000308 (46/149288, GnomAD_genomes) | delG=0.0065 (47/7234, Korea) | Pathogenic : PVS1, PM3_Very Strong, PS3_Moderate, BS1 |
| SB541-1013 | *GJB2* | [NM_004004.5] c.235del | [NP_003995.2] p.Leu79Cys*fs*Ter3 | AR | Chr13:20763485 AG>A rs80338943 | heterozygote | 34.00 | NA | delG=0.0001834 (257/1401430, GnomAD_exomes) delG=0.000308 (46/149288, GnomAD_genomes) | delG=0.0065 (47/7234, Korea4K) | Pathogenic : PVS1, PM3_Very Strong, PS3_Moderate, BS1 |
|  |  | [NM_004004.5] c.176_191del | [NP_003995.2] p.Gly59Ala*fs*Ter18 | AR | Chr13:20763529 GCACACGTTCTTGCAGC>G  rs750188782 | heterozygote | No match in CADD v1.7 | NA | delACACGTTCTTGCAGCC=0.0000143 (20/1400898, GnomAD_exomes) | delACACGTTCTTGCAGCC=0.0001 (1/7234, Korea4K) | Pathogenic : PVS1, PM3_Very Strong, PM2 |
| SB430-834 | *POU3F4* | Large genomic deletion affecting *POU3F4* |  | AD | ChrX:(?_82763298)_82767128_?) del | hemizygote | NA | NA | absent | absent | Pathogenic : PVS1, PM2, PP4 |
| SB606-1104 | *POU3F4* | Large genomic inversion affecting *POU3F4* enhancer |  | AD | chrX:51,812,227~83,172,187 inversion [GRCh38] | hemizygote | NA | NA | absent | absent | Pathogenic : PVS1, PM2, PP4 |
| SB332-653 | *POU3F4* | Large genomic deletion affecting *POU3F4* enhancer |  | AD | ChrX:(?_82763298)_82767128_?) del | hemizygote | NA | NA | absent | absent | Pathogenic : PVS1, PM2, PP4 |
| SB508-972 | *MYO15A* | [NM_016239.4] c.1185dup | [NP_057323.3] p.Glu396Arg*fs*Ter36 | AR | Chr17:18023292 A>AC dbSNP ID : absent | heterozygote | 24.70 | NA | absent | absent | Pathogenic : PVS1, PM2, PM3_Supporting |
|  |  | [NM_016239.4] c.2419C>T | [NP_057323.3] p.Gln807Ter | AR | Chr17:18024533 C>T  rs1233145763 | heterozygote | 36.00 | NA | T=0.0000567 (73/1287596, GnomAD_exomes) T=0.000020 (3/148218, GnomAD_genomes) | T=0.0001 (1/7098, Korea4K) | Pathogenic : PVS1, PM2, PM3_Supporting |
| SB565-1043 | *MYO15A* | [NM_016239.4] c.1185dup | [NP_057323.3] p.Glu396Arg*fs*Ter36 | AR | Chr17:18023292 A>AC dbSNP ID : absent | heterozygote | 24.70 | NA | absent | absent | Pathogenic : PVS1, PM2, PM3_Supporting |
|  |  | [NM_016239.4] c.9787+1G>A |  | AR | Chr17:18067153 G>A  rs769937488 | heterozygote | 32.00 | NA | absent | absent | Pathogenic : PVS1, PS1, PM2 |
| SB290-582 | *MYO6* | [NM_004999.4] c.667G>A | [NP_004990.3] p.Gly223Arg | AD | Chr6:76550946 G>A dbSNP ID : absent | heterozygote | 28.20 | 0.923 | absent | absent | Uncertain Significance : PM2, PP1, PP3 |
| SB651-1164 | *EYA1* | [NM_000503.5] c.1117_1118del | [NP_000494.2] p.His373Phe*fs*Ter4 | AD | Chr8:72156859 ATG>A rs886039674 | heterozygote | No match in CADD v1.7 | NA | absent | absent | Pathogenic : PVS1, PS2, PM2, PP4 |
| SB1385-2165 | *TECTA* | [NM_005422.4] c.5597C>T | [NP_005413.2] p.Thr1866Met | AD | Chr11:121038773 C>T rs140236996 | heterozygote | 29.70 | 0.761 | T=0.000007 (1/149156, GnomAD_genomes) | absent | Pathogenic : PS1, PP1_Strong, PM2, PS4_Moderate, PP3 |
| SB758-1340 | *TMPRSS3* | [NM_001256317.3] c.1210dup | [NP_001243246.1] p.Leu404Pro*fs*Ter63 | AR | Chr21:43795958 A>AG dbSNP ID : absent | heterozygote | No match in CADD v1.7 | NA | absent | absent | Pathogenic : PVS1, PM2, PM3_Supporting |
|  |  | [NM_001256317.3] c.346G>A | [NP_001243246.1] p.Val116Met | AR | Chr21:43808612 C>T rs200090033 | heterozygote | 23.80 | 0.695 | T=0.000040 (6/149162, GnomAD_genomes) | absent | Uncertain Significance : PM2, PM3_Supporting |
| SB518-984 | Not identified |  |  |  |  |  |  |  |  |  |  |
| SB711-1269 | Not identified |  |  |  |  |  |  |  |  |  |  |
| SB602-1100 | Not identified |  |  |  |  |  |  |  |  |  |  |
| SB718-1281 | Not identified |  |  |  |  |  |  |  |  |  |  |
| SB809-1408 | Not identified |  |  |  |  |  |  |  |  |  |  |
| SB388-752 | Not identified |  |  |  |  |  |  |  |  |  |  |

Abbreviations: AR, autosomal recessive; GMAF, global minor allele frequency; NA, not available; ACMG, the American College of Medical Genetics and Genomics; AMP, the Association for Molecular Pathology.

Web resources: CADD: Combined Annotation Dependent Depletion (https://cadd.gs.washington.edu/),REVEL: Rare Exome Variant Ensemble Learner (https://sites.google.com/site/revelgenomics/),KOVA: Korean Variant Archive for a reference database of genetic variations in the Korean population (https://www.kobic.re.kr/kova/) ,gnomAD: The Genome Aggregation Database (https://gnomad.broadinstitute.org/).
