# Supplementary material for: Comparison of One-Year auditory rehabilitation outcomes by etiology in pediatric patients with bilateral severe hearing loss (70–90 dB): enlarged vestibular aqueduct vs. Other causes
Source: Eur Arch Otorhinolaryngol. 2025 Sep 18;283(1):149–56. doi: 10.1007/s00405-025-09649-6 (PMC12904901; doi:10.1007/s00405-025-09649-6)
Supplement: Supplementary file 4 — (DOCX 16.3 KB) [file 405_2025_9649_MOESM4_ESM.docx]

**Supplementary table 4.** A comparison of each speech performance results between pre- and post-treatment is indicated. A Wilcoxon signed rank test was performed between pre- and post- treatment period and a *p*-value of less than 0.05 was reported as statistically significant. Only the EVA group demonstrated significantly better expressive language outcomes compared to the non-EVA group after one year of hearing aid rehabilitation

|  | **Group** | **Pre-treatment** | **Post-treatment** | ***p*-value** |
| --- | --- | --- | --- | --- |
| Hearing threshold  (dB) | EVA | 76.1 ± 7.1 | 82.3 ± 13.2 | 0.073 |
|  | Non-EVA | 75.1 ± 5.7 | 74.6 ± 12.8 | 0.760 |
| CAP | EVA | 2.6 ± 1.5 | 4.3 ± 1.2 | 0.001 |
|  | Non-EVA | 3.0 ± 1.7 | 4.4 ± 1.1 | 0.001 |
| Receptive  (age, month) | EVA | 10.6 ± 7.6 | 20.8 ± 8.7 | 0.001 |
|  | Non-EVA | 15.4 ± 17.9 | 25.9 ± 15.5 | 0.000 |
| Receptive  (percentile) | EVA | 23.4 ± 22.6 | 33.3 ± 32.3 | 0.277 |
|  | Non-EVA | 24.1 ± 31.9 | 27.9 ± 30.1 | 0.551 |
| Expressive  (age, month) | EVA | 11.25 ± 7.0 | 19.6 ± 9.7 | 0.002 |
|  | Non-EVA | 12.8 ± 9.6 | 18.5 ± 6.3 | 0.000 |
| Expressive  (percentile) | EVA | 26.0 ± 22.1 | 41.8 ± 30.9 | 0.069 |
|  | Non-EVA | 27.9 ± 28.1 | 20.4 ± 28.8 | 0.116 |

Abbreviations: CAP, categories of auditory performance; EVA, enlarged vestibular aqueduct.
